# Understanding diversity–stability relationships: towards a unified model of portfolio effects

**DOI:** 10.1111/ele.12019

**Published:** 2012-10-24

**Authors:** Loïc M Thibaut, Sean R Connolly, Fangliang He

**Affiliations:** 1 School of Marine and Tropical Biology, ARC Centre of Excellence for Coral Reef Studies, James Cook UniversityTownsville, QLD 4811, Australia

**Keywords:** Diversity–stability relationship, evenness, insurance hypothesis, mean-variance scaling, overyielding, population stability, population synchrony, portfolio effect, statistical averaging

## Abstract

A major ecosystem effect of biodiversity is to stabilise assemblages that perform particular functions. However, diversity–stability relationships (DSRs) are analysed using a variety of different population and community properties, most of which are adopted from theory that makes several restrictive assumptions that are unlikely to be reflected in nature. Here, we construct a simple synthesis and generalisation of previous theory for the DSR. We show that community stability is a product of two quantities: the synchrony of population fluctuations, and an average species-level population stability that is weighted by relative abundance. Weighted average population stability can be decomposed to consider effects of the mean-variance scaling of abundance, changes in mean abundance with diversity and differences in species' mean abundance in monoculture. Our framework makes explicit how unevenness in the abundances of species in real communities influences the DSR, which occurs both through effects on community synchrony, and effects on weighted average population variability. This theory provides a more robust framework for analysing the results of empirical studies of the DSR, and facilitates the integration of findings from real and model communities.

## Introduction

In nature, many species' ecological roles are essential for ecosystem functioning or for the provision of ecosystem services to human societies (Lawton 1994; Worm *et al*. 2006; Cardinale *et al*. 2012). For example, species that forage for pollen or nectar facilitate reproduction in the plants on which they forage, a function that is essential for the maintenance of plant populations, including in agricultural ecosystems (McGregor 1976; Hoehn *et al*. 2008). On coral reefs, grazing by fishes helps to maintain healthy, coral-dominated reefs (Bellwood *et al*. 2004). One essential service provided by biodiversity is to stabilise the overall abundance of an assemblage of organisms that provides a particular ecosystem service or function, thereby making it less vulnerable to fluctuations in the abundances of individual populations. This phenomenon, or components of it, has been characterised using a variety of terms (e.g. statistical averaging, portfolio effect, covariance effect, insurance hypothesis and stabilising effect: Doak *et al*. 1998; Tilman *et al*. 1998; Yachi & Loreau 1999; Loreau 2010). For simplicity, we here refer to the relationship between the number of populations and temporal stability of total community abundance as the ‘diversity–stability relationship’, or DSR, and the tendency for DSRs to be positive (i.e. for stability to increase with diversity), as the ‘portfolio effect’. This definition of the latter term is consistent with its use in other disciplines, such as finance (Markowitz 1952, 1987), and with its original use in the context of the DSR (Tilman *et al*. 1998; *contra* Tilman 1999).

In both model and experimental communities, stability is typically taken to be inversely related to the coefficient of variation of some measure of ecosystem function, such as total community abundance. The DSR is the relationship between this measure of stability, and diversity (here defined as the number of constituent populations). Usually, diversity is quantified as species richness, but communities can be stabilised by diversity at other levels of organisation as well, such as functional groups (Bai *et al*. 2004) or number of phenotypes within populations (Norberg *et al*. 2001). Various proposed statistical formalisms for the DSR have suggested that, at least in principle, it may be positive or negative (Tilman 1999; Lhomme & Winkel 2002). However, stochastic competition models have consistently found portfolio effects (e.g. Lehman & Tilman 2000; Ives & Hughes 2002; Loreau & de Mazancourt 2008). Similarly, two decades of experimental research into DSRs indicates that portfolio effects are overwhelmingly present, but that their strength and magnitude varies considerably (Campbell *et al*. 2011). However, inverse portfolio effects, where stability decreases with diversity, also can occur in nature (DeClerck *et al*. 2006; Yang *et al*. 2011).

Several community properties have been identified as important determinants of the portfolio effect. Four that have received particular attention are asynchrony in population fluctuations, evenness of abundance, effects of diversity on total community abundance and the way in which temporal variability in abundance scales with its mean (Cottingham *et al*. 2001). Firstly, theoretical studies indicate that portfolio effects should strengthen as asynchrony in the fluctuations of a community's constituent populations increases (Doak *et al*. 1998; Loreau 2010). Despite its importance in diversity–stability relationships, however, there is no consensus about how asynchrony should be measured, or about how it contributes to the DSR. A variety of metrics have been proposed, including coefficients of pairwise correlations of species' fluctuations in abundance (Doak *et al*. 1998), summed species-level variances and covariances (Tilman 1999) and total community variance relative to that of a perfectly synchronous community (Loreau & de Mazancourt 2008). All of these metrics are still used in empirical studies (e.g. Mikkelson *et al*. 2011; Roscher *et al*. 2011; Thibaut *et al*. 2012). Secondly, models of the DSR also predict that the portfolio effect will be stronger, where evenness of mean abundance among populations is greater (Doak *et al*. 1998; Lhomme & Winkel 2002): when evenness is very low, the rarest species make a limited contribution to overall stabilisation of function at the community level, compared with when evenness is high. However, researchers have found positive relationships between evenness and stability (Mikkelson *et al*. 2011), no relationship (Isbell *et al*. 2009) and even negative relationships (van Ruijven & Berendse 2007), leading to calls for the development of theory to better understand how evenness affects community stability (Grman *et al*. 2010; Mikkelson *et al*. 2011). Thirdly, mean community biomass often increases with increasing diversity (Duffy 2009; Cardinale *et al*. 2012), a phenomenon sometimes termed ‘overyielding’ (Tilman 1999), and several empirical studies have identified overyielding as a mechanism driving the DSR (Tilman *et al*. 2006; Isbell *et al*. 2009; Hector *et al*. 2010). Finally, there is a well-known tendency for the temporal variance in population abundance to exhibit a power-law relationship with the mean (Taylor 1961). Theoretical studies have suggested that stability should increase as the value of the exponent of this mean-variance scaling relationship increases above unity (Tilman *et al*. 1998; Tilman 1999). However, some experimental studies have found contrary results (Valone & Hoffman 2003; van Ruijven & Berendse 2007; Yang *et al*. 2011).

A comprehensive understanding of the combined effects of synchrony, overyielding, mean-variance scaling and evenness on the diversity–stability relationship has been hampered by the need to make idealised assumptions about some of these phenomena when investigating effects of others. For example, to examine the effect of evenness, Doak *et al*. (1998) assumed that all between-species correlations are equal, and that total community size is independent of diversity (no overyielding). Tilman's (1999) framework assumes perfect evenness (all species' mean abundances are equal), and independence of species fluctuations (all *ρ*_*ij*_ = 0), to examine the effect of overyielding. Similarly, community-dynamic approaches have made strong symmetry assumptions (e.g. all species have the same intrinsic growth rates, carrying capacities, competition coefficients and between-species correlations in responses to environmental fluctuations: Ives & Hughes 2002; Loreau 2010). These assumptions have come under increasing criticism, particularly in empirical studies that have obtained anomalous results (such as ‘inverse’ portfolio effects, where communities become less stable as diversity increases) under conditions where particular simplifying assumptions are violated (e.g. Valone & Hoffman 2003; Steiner *et al*. 2005; van Ruijven & Berendse 2007; Grman *et al*. 2010; Yang *et al*. 2011).

An additional challenge to understanding the DSR is teasing apart the factors that drive the relationship between community stability, and stability of the individual populations that constitute the community. In a meta-analysis, Campbell *et al*. (2011) found strongly bimodal responses of population stability with diversity: some studies find that diversity stabilises populations, while a comparable number of studies find that diversity destabilises populations. Because either of these contrasting population-level responses may occur in assemblages exhibiting portfolio effects at the community level, clarifying the relationship between population stability and overall community stability has been identified as a critical knowledge gap in our understanding of the DSR (Vogt *et al*. 2006; Campbell *et al*. 2011).

To generalise the theory that we use to understand the DSR, and to place earlier theoretical and empirical findings in a broader context, we here synthesise key elements of previous approaches (e.g. Doak *et al*. 1998; Tilman 1999; Loreau 2010), to produce a simple model of portfolio effects that makes explicit how community stability relates to the stability of a community's constituent populations, and in turn how asynchrony, overyielding, mean-variance scaling and evenness influence this relationship. Analysis of this model reveals that the DSR is the product of a synchrony effect and a weighted average population variability effect, a simple expression that is robust to the presence or absence of overyielding, and to differences in means or variances of species abundances (i.e. arbitrary violation of the evenness assumption). Weighted average population variability can be further decomposed into an overyielding-related effect and a single-species variability effect. This synthetic framework clarifies the sometimes counter-intuitive ways that evenness can affect the DSR, and helps to explain apparent inconsistencies among alternative statistical frameworks, empirical studies of the DSR and theoretical studies based on analysis of community-dynamic models. It also suggests some additional assumptions common to DSR theory that are likely to be violated in nature, but whose effects on the DSR have received little or no attention to date.

## Towards a Unified Model of Portfolio Effects

Following previous theory for the portfolio effect (e.g. Doak *et al*. 1998; Tilman 1999; Loreau 2010), we here treat species abundances as stationary random variables (i.e. abundances fluctuate over time, with a fixed mean and variance). Thus, a community of *n* species can be described with a vector of mean species abundances, **m**_*n*_ and a variance–covariance matrix of abundances, **V**_*n*_: 

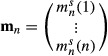
1a

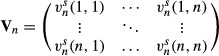
1bwhere 

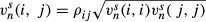
1c

In eqn 1c, and throughout this article, we use the superscript *c* to designate community level quantities and the superscript *s* for species-level quantities. *n* is the number of species in the community, *m* denotes mean abundances and *v* denote variances and covariances. Thus, 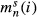 denotes the mean abundance of species *i*, 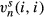 the variance (over time) of abundance of species *i,* and 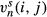 the temporal covariance of abundances of species *i* and *j*, in a community of *n* species. By definition, the covariances depend on the species-specific variances, and a coefficient, *ρ*_*ij*_, characterising the temporal correlation between the abundances of the two species.

### Measuring asynchrony

Of particular interest in analyses of the portfolio effect has been the development of measures of community synchrony. In early study, community synchrony was defined using the correlation coefficients in eqn 1, which were assumed to be the same for all pairs of species in the community (*ρ*_*ij*_ *= ρ* for all *i,j*) (Doak *et al*. 1998; Tilman 1999). As, in real communities, correlation coefficients will differ between different pairs of species, based on the idiosyncratic characteristics that determine their interactions and responses to environmental fluctuations, most empirical analyses rely on the mean of the correlation coefficients, 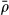 (Valone & Barber 2008; Thibaut *et al*. 2012), which is bounded in the range 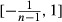. A problem with this approach is that, in real communities, species may differ substantially in their variances, so some between-species correlations are likely to be more important to overall community stability than others. Consequently, two communities with the same mean correlation coefficient could differ substantially in their synchrony (see Appendix S1 in Supplementary Information for an example).

An alternative approach to measuring synchrony considers the sum of the species-level variances (diagonal elements of eqn 1b) and the sum of the between-species covariances (off-diagonal elements of eqn 1b). Tilman (1999) argued that these two quantities measure different drivers of asynchrony: the former a ‘portfolio effect’ – the benefit of diversity due to statistical averaging (a narrower definition of the term than used in this article) – and the latter a ‘covariance effect’, which represents the stabilising effect of compensatory interactions (e.g. the tendency for a species to increase in abundance from competitive release, when another species decreases). However, while there is still some disagreement in the literature about the utility of summed covariances as an indicator of compensatory interactions, there is now broad consensus that summed variances and covariances do not partition statistical averaging and compensatory interaction effects (Ives & Carpenter 2007; Houlahan *et al*. 2008; Loreau & de Mazancourt 2008; Ranta *et al*. 2008).

More recently, Loreau & de Mazancourt (2008) proposed quantifying community synchrony using the statistic: 

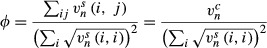


(also see Loreau 2010). Here, the scalar 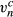 indicates the variance of total community abundance for a community of *n* species, which, by definition, is the sum of all elements of the community variance–covariance matrix (the summed variances plus the summed covariances). The denominator is the variance of a hypothetical community with the same species-level variances, but in the presence of perfect synchrony (Loreau & de Mazancourt 2008). One advantage of *ϕ*, hereafter termed the ‘synchrony index’, is that it makes no assumptions about the particular distribution of values for the pairwise correlation coefficients. This is because the off-diagonal elements of the community variance–covariance matrix influence *ϕ* only through their combined effect on the total community variance in abundance, 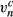, which can be measured directly in the aggregate (i.e. without separate estimation of pairwise covariances). *ϕ* is also normalised, independent of diversity: it always varies between zero (when total community abundance is constant), and one (when fluctuations are perfectly synchronous). Finally, in contrast to the mean correlation coefficient, it explicitly incorporates the effects of unequal species-level variances on synchrony (see Appendix S1).

### Unifying population and community variability

**Figure 1:**
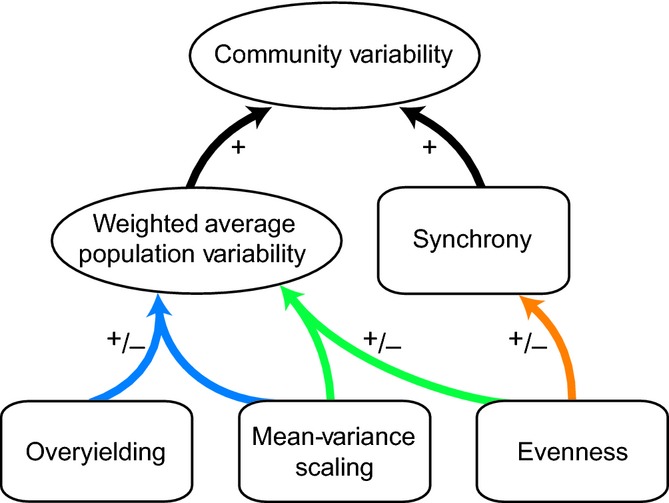
Schematic illustrating how population and community variability (ellipses) are influenced by the four determinants of the DSR highlighted in the Introduction: synchrony of population fluctuations, evenness, overyielding and the way variance in population fluctuations scales with the mean (rectangles). Arrows merge where an effect arises from an interaction between two determinants. ‘+’ indicates that an effect is positive (e.g. community variability increases as synchrony increases), and ‘+/−’ indicates that an effect may be either positive or negative.

We can derive a very general relationship for the relationship between population and community variability by taking advantage of the synchrony index, re-arranging eqn 2 and rescaling our measure of community variability from total variance to CV: 

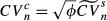
where 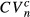 is the coefficient of variation of total community abundance, for a community of *n* species, and 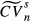 is the average species-level coefficient of variation for a community of *n* species, weighted by species' relative mean abundance: 

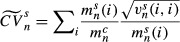
(see Appendix S2 in Supplementary Information for derivation). 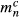 is the temporal mean of total community abundance (i.e. the sum of species-level mean abundances). Equation 3 shows that the dimensionless community variability in abundance is completely determined by the weighted average species-level coefficient of variation, and the synchrony index *ϕ* (Fig. 1, black arrows). In eqn 3, dimensionless population and community variability are linearly proportional to one another, with a constant of proportionality that depends on how synchronous the fluctuations of different species are. When fluctuations are highly synchronous (*ϕ*~1), community variability tracks population variability. When fluctuations are less synchronous (*ϕ* is smaller), population variability is damped at the community level. The fact that population variability is a weighted average in eqn 3 indicates that the variability of more abundant populations make larger contributions to overall community variability.

Equation 3 is much more general than previous DSR models. In particular, it makes no assumptions about evenness of mean abundances, about the distribution of variances or correlation coefficients in the covariance matrix, or about the direct ecological interactions or responses to environmental fluctuations that influence those variances and correlation coefficients. Note that, because *ϕ* ≤ 1, community variability is never greater than population variability.

To examine more specifically the role of overyielding and mean-variance scaling, we extend eqn 3 by making two further assumptions that have reasonably broad empirical support. Firstly, we assume that temporal variances in species' population sizes scale with their means according to Taylor's (1961) power law: 

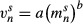
where *a* and *b* are coefficients relating mean and variance of abundance. Secondly, both species and community mean abundance may vary as a function of diversity. We model this phenomenon using Tilman's (1999) functional form for this relationship, modified to allow unequal mean abundances: 

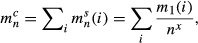
where *m*_1_(*i*) is the abundance of species *i* in monoculture, and *x* drives how the total abundance of the community changes with diversity. If *x* = 1, the abundance of the total community is fixed, independent of diversity, as is assumed in many theoretical studies of the DSR (*sensu* Doak *et al*. 1998; Ives *et al*. 1999; Loreau 2010). ‘Overyielding’ occurs whenever *x* < 1: the mean of total community abundance increases with diversity. If 0 < *x* < 1, this increasing community abundance is accompanied by decreases in mean species abundances with diversity; if *x* = 0, mean species abundances are independent of diversity; and if *x* < 0, mean species abundances actually increase with diversity. Finally, if *x* > 1, mean community abundance and mean species abundances both decrease with diversity (underyielding).

Incorporating eqns 7 and 8 into eqn 3, we can extend our framework to explicitly include the effect of overyielding on the DSR: 

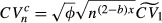
(see Appendix S3 in Supplementary Information for derivation). This essentially sub-divides species-level population variability into two components: an ‘average single-species variability’ term, 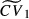 which represents species’ weighted average CV in monoculture, and a ‘mean-abundance effect’, 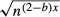, which characterises how 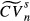 changes with diversity as a consequence of associated systematic changes in mean abundance (Fig. 1, blue arrows). Equation 9 generalises Tilman's (1999) model considering the effect of overyielding on the portfolio effect, which assumes that all species have the same mean abundance, and species' fluctuations in abundance are uncorrelated with one another. Similarly, it can be considered a generalisation of eqn 5.8 in Loreau (2010), who considered the special case of no overyielding and perfect evenness. Note that, if the mean-abundance effect increases with diversity, then changes in mean species abundances associated with increasing diversity tend to be destabilising at both population and community levels. In contrast, if the mean-abundance effect decreases with diversity, then changes in mean species abundance associated with increasing diversity are stabilising at both population and community levels.

### Synchrony, overyielding, evenness and the portfolio effect

Equation 3 makes explicit how portfolio effects arise from changes in synchrony and population variability with species richness. To understand how synchrony is likely to change with diversity, it is helpful to consider the relationship between *φ* and the mean correlation coefficient, 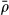, derived by Loreau (2010): 

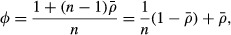


**Figure 2:**
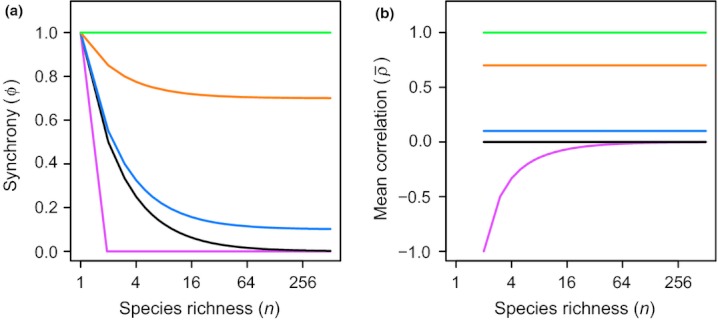
Illustration of relationship between diversity-dependence of (a) the synchrony index, *ϕ*, and (b) the mean correlation coefficient, 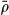, under the assumption of equal population variances. The green line shows the limiting case of perfect synchrony (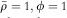). The magenta line shows the limiting case of perfect asynchrony 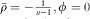 whenever *n* > 1). For the orange, blue and black lines, 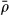 is constant, independent of diversity (at 0.7, 0.1, and 0 respectively), so *ϕ* is calculated from eqn 8 using the specified value of 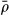. Because 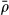 is only defined for *n* > 1, the lines in panel (b) commence at *n* = 2. Note that species richness is plotted on a logarithmic scale.

Equation 8 holds only for the special case when all species have the same variances, but is still useful for thinking about the implications of different community structures for the diversity-dependence of synchrony. For instance, in the limiting case of perfect synchrony, 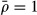 and *ϕ* = 1, regardless of diversity (Fig. 2, green line). Conversely, for perfect asynchrony, 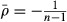, and *ϕ* = 0 everywhere (except in monoculture, where *ϕ* = 1) (Fig. 2, magenta line). For the special case of a community of non-interacting species, fluctuations in abundance between species are correlated only due to similarities in their responses to environmental fluctuations, so pairwise correlation coefficients are constant, independent of diversity (Ives *et al*. 1999). If 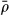 is small, *ϕ* decreases strongly with diversity (Fig. 2, black and blue lines), while if 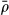 is large, then *ϕ* is less strongly diversity-dependent (Fig. 2, orange line). This tendency for *ϕ* to decline asymptotically with diversity also occurs in the presence of competition (see Appendix S4 in Supplementary Information), and there are good reasons to expect this tendency to be common in nature. Firstly, *ϕ* = 1 in monoculture, and must decline from this value as diversity increases if species are not perfectly positively correlated. Secondly, as diversity becomes large, each additional species makes a progressively smaller marginal contribution to the overall mean correlation coefficient, implying that changes in 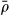 (and thus, by eqn 8, *ϕ)* will become smaller and smaller as diversity increases.

**Figure 3:**
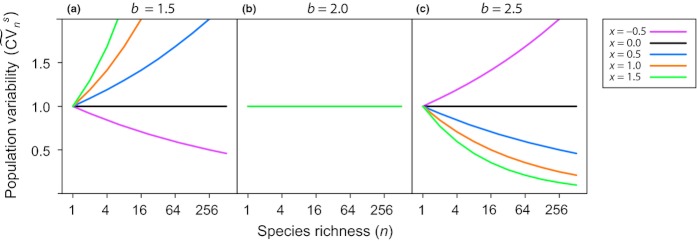
Illustration of the diversity-dependence of the mean-abundance effect for mean-variance scaling exponents of (a) *b* = 1.5, (b) *b* = 2, and (c) *b* = 2.5. Results are qualitatively identical to (a) whenever *b* < 2, and to (c) whenever *b* > 2. The different-coloured lines show the mean-abundance effect for different values of the overyielding coefficient, *x*, as indicated in the figure. Note that all lines are super-imposed when *b* = 2. Species richness is plotted on a logarithmic scale.

Although the influence of overyielding on population variability has been investigated previously, these studies have tended to focus on the ranges 0 < *x* < 1 and 1 < *b* < 2 (e.g. Lhomme & Winkel 2002). In this range, the mean-abundance effect causes population variability to increase with diversity (Fig. 3a, blue line). The focus on 1 ≤ *b* ≤ 2 was likely due to a belief that exceptions to this range are rare (e.g. Kilpatrick & Ives 2003). However, a growing number of studies report scaling exponents > 2 (e.g. Valone & Hoffman 2003; Vogt *et al*. 2006; van Ruijven & Berendse 2007). Similarly, a focus on 0 ≤ *x* ≤ 1 makes sense for manipulations of diversity under fixed environmental conditions, where *x* reflects mainly the combined effects of competition and niche partitioning (Tilman 1999; Lehman & Tilman 2000). However, in nature, diversity often covaries with environmental conditions that influence mean abundance in other ways, and DSR studies along natural gradients have found both cases of underyielding (*x* > 1; Yang *et al*. 2011), and cases where mean species abundance actually increases with diversity (*x* < 0: Valone & Hoffman 2003). Considering this broader range of parameter values, the mean-abundance effect can be seen to have both positive and negative effects on population variability (Fig. 3). The direction of the mean-abundance effect depends on whether mean species abundance decreases with species richness or not (*x* > 0 or *x* < 0), and whether species-level variances scale less than or more than quadratically with the mean (*b* < 2 or *b* > 2). Specifically, if mean species abundance decreases with species richness (*x* > 0), then the mean-abundance effect is destabilising at the population level when variance scales less than quadratically with mean species abundance (*b* < 2), and stabilising when *b* > 2 (compare blue, orange and green lines in Fig. 3a,c). If mean species abundance increases with species richness (*x* < 0), then mean-variance scaling has the opposite effect (compare magenta lines in Fig. 3a,c).

**Figure 4:**
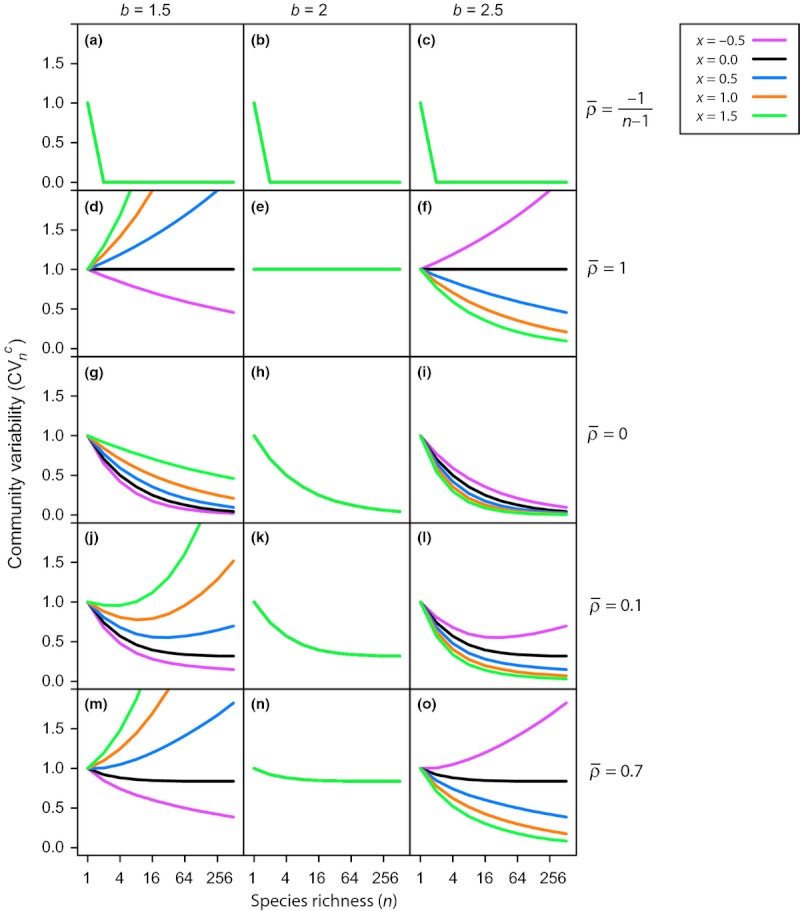
Combined influences of synchrony and the mean-abundance effect on community variability. The left column of panels shows *b* = 1.5 (cf. Fig. 3a), the middle column *b* = 2 (cf. Fig. 3b), and the right column *b* = 2.5 (cf. Fig. 3c). Rows of panels incorporate different models of synchrony from Fig. 2 (calculated from eqn 8 using the values of the mean correlation coefficient to the right of each row on the panel above). Coloured lines represent different values of the overyielding coefficient, *x*, as in Fig. 3 (as indicated by the legend on the panel above). Note that all lines are super-imposed when *b* = 2, or 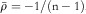. Species richness is plotted on a logarithmic scale.

Figure illustrates how the DSR may be influenced by the interaction between community synchrony and the mean-abundance effect. Several non-intuitive results are worth highlighting. Firstly, a portfolio effect can be apparent even when synchrony is perfect, if population variability decreases with species richness (Fig. 4f, blue, orange and green lines). Secondly, Fig. 4 shows that inverse portfolio effects are possible (e.g. Fig. 4m). Thirdly, when population variability and synchrony act in opposite directions, non-monotonic DSRs can be produced, for which community variability initially decreases with species richness, then increases. For instance, when 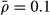, independent of diversity, *ϕ* decreases asymptotically towards 0.1 as richness increases (eqn 8). Thus, its response to diversity may dominate the DSR at low diversity, while population variability dominates at high diversity (compare blue line in Fig. 2a with orange lines in Figsa and 4j).

**Figure 5:**
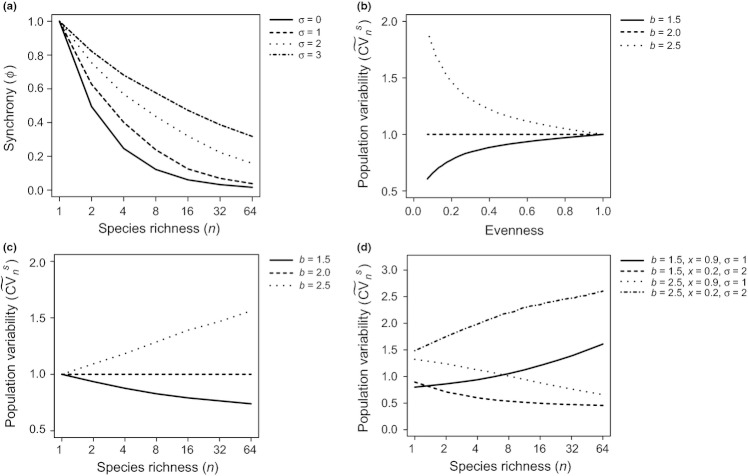
Illustration of effects of unevenness on the DSR. In all panels, species abundances are drawn from a lognormal distribution with mean of log abundance μ = 1, and mean-variance scaling coefficient *a* = 1. (a) Diversity-dependence of synchrony for different levels of unevenness, generated using the specified standard deviation of log abundance, *σ*. For all curves, *b* = 2 and correlation coefficients were assigned randomly as described in the text. (b) 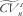 as a function of evenness [Communities were simulated using 0 ≤ σ ≤ 3, and evenness quantified using the index *E*_*var*_ (Smith & Wilson 1996)]. (c) 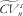 vs. diversity, illustrating the effect of *b*. For all lines, *σ* = 2 and *x* = 0. (d) 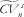 vs. diversity, illustrating the interaction between evenness and mean-abundance effects. Note the log-scale for species richness.

Considering the effect of unevenness in light of eqns 3 and 7 indicates that it may actually increase or decrease community variability, and may increase or decrease the strength of the portfolio effect, depending on its effects on the synchrony index, *ϕ*, and on species population variability, 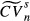. The effect of evenness on *ϕ* (Fig. 1, orange arrow) depends on the how the population sizes of the different species fluctuate relative to one another (i.e. on the structure of the covariance matrix, **V**_**n**_ [eqn 1b]). Previous consideration of the effect of evenness on the DSR has assumed, implicitly or explicitly, that the populations of all species pairs are equally correlated (all *ρ*_*ij*_ *= ρ*: see, e.g. Doak *et al*. 1998). However, in the general case where the *ρ*_*ij*_ differ, the effect of unevenness is more contingent. For example, consider an assemblage in which population fluctuations of most species are highly synchronous, except for one species, whose fluctuations are strongly negatively correlated with all the other species (as in Appendix S1). For this community, the portfolio effect will be maximised when this latter species is disproportionately abundant (or, more precisely, contributes disproportionately to the total community variance). Moreover, non-intuitive effects of evenness can emerge even when correlation coefficients are independent of relative abundance. For example, in Fig. 5a, we generated hypothetical communities by drawing species' mean abundances at random from a lognormal distribution, and we assigned pairwise correlation coefficients at random with respect to abundance such that the expected mean correlation coefficient is zero, regardless of evenness or diversity. [We achieved the latter by exploiting a hyperspherical parameterisation of the correlation matrix (Pinheiro & Bates 1996). By drawing each parameter from a uniform distribution on [0, π], we sample from the entire universe of possible correlation matrices where, on average, the mean correlation coefficient is zero.] When there is perfect evenness, *ϕ* for the randomly assembled community is identical to the theoretical prediction (eqn 8 with 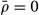). As evenness decreases, synchrony increases, consistent with the hypothesis that unevenness is destabilising at the community level. However, the same asymptotic value is approached at high diversity, so the effect of this is to cause synchrony to decrease more gradually with diversity when unevenness is higher (Fig. 5a).

Evenness can also influence weighted average population variability, 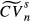 but its qualitative effect depends upon the nature of mean-variance scaling (Fig. 1, green arrows). Specifically, as evenness decreases, 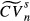 decreases when species' population variability scales less than quadratically with the mean (*b* < 2), and increases when *b* > 2 (Fig. 5b). This is because weighted average population variability becomes progressively less dominated by the more abundant species as evenness increases. In particular, when *b* < 2, CV decreases with mean abundance. As evenness decreases, the most abundant species occupy a progressively larger fraction of the community, and thus, population variability becomes progressively more dominated by these low-variability populations.

This relationship between evenness and population variability implies that unevenness can alter the way in which population variability changes with species richness, even when species are assembled randomly into communities with respect to their mean abundances. It is easiest to understand this effect by considering first the special case, when species' mean abundances are independent of diversity (*x* = 0, so the mean-abundance effect in eqn 7 is unity). In this case, unevenness tends to cause population variability to decrease with diversity when *b* < 2, and to increase when *b* > 2 (Fig. 5c). We interpret this result as follows. As diversity increases, the likelihood of the assemblage, by chance, containing a species with very high mean abundance increases. The populations of these highly abundant species will be more or less stable than the populations of species with average mean abundance, depending on whether *b* < 2 or *b* > 2 respectively. Of course, there is also a progressively greater likelihood of including species with unusually small mean abundances as diversity increases. However, because 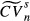 is a weighted average, the effect of sampling further out in the abundant tail of the distribution outweighs the countervailing effect of sampling further out in the rare tail. This interpretation is supported by simulations using a wide variety of shapes of species-abundance distributions (including symmetrical, left and right-skewed distributions), and by the fact that in contrast to 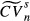 unweighted population variability exhibits no trends with diversity, when *x* = 0, regardless of *b* (results not shown).

The nature of the interaction between unevenness and the mean-variance scaling parameter *b* implies that when species' abundances differ (i.e. unevenness is present) and species’ mean abundances tend to decrease with species richness (*x* > 0), mean-variance scaling may actually act in countervailing ways along a diversity gradient. For instance, when *x* > 0 and *b* < 2, the mean-abundance effect is destabilising at the population level, but the effect of unevenness is stabilising (e.g. compare Fig. 3a, blue line, and Fig. 5c, solid line), whereas the opposite occurs when *b* > 2 (e.g. Fig. 3c, blue line, and Fig. 5c, dotted line). This makes sense: increasing unevenness will tend to make the most abundant species – which dominate the weighted average population variability – increasingly more abundant relative to the average species mean abundance. Consequently, populations will be more stable if *b* < 2 and less stable if *b* > 2. Conversely, *x* > 0 will tend to reduce all species’ mean abundances as diversity increases, so populations will become less stable if *b* < 2, and more stable if *b* > 2. Thus, whether population variability is stabilised or destabilised by unevenness depends upon the magnitude of the overyielding parameter, *x*, relative to the among-species variance in mean abundances (i.e. the extent of unevenness), and on whether *b* < 2 or *b* > 2 (Fig. 5d).

Of course, along natural diversity gradients, species may not be added at random with respect to their mean abundances, with implications for how population variability changes with species richness. For example, species’ colonisation abilities are sometimes hypothesised to be negatively correlated with their resource-use efficiency, (and thus population density at equilibrium: e.g. Tilman & Downing 1994). In such cases, succession would be expected to commence with species that have coloniser strategies, low resource-use efficiency and thus low equilibrium abundance, and to progress by adding poorer colonisers that have greater resource-use efficiency and higher equilibrium abundances (Tilman & Downing 1994). As these conditions imply that species tend to be added to communities in order of progressively increasing mean abundances, weighted average population variability would tend to decrease with species richness if *b* < 2 (because species with higher mean abundances have lower CV when *b* < 2, and these species are increasingly represented as species richness increases). Conversely, weighted average population variability would increase with species richness under these conditions if *b* > 2.

## Diversity and Stability in Nature

The model in eqn 3 shows that there are two key elements to making explicit the relationship between population and community variability. The first is to define community synchrony in terms of the synchrony index *ϕ* of Loreau & de Mazancourt (2008). The second is to measure population variability as a weighted average across species. The resulting relationship is extremely general. It holds regardless of extent or nature of unevenness of abundances among species, and regardless of the pattern of variances or covariances of species’ abundances. Thus, it is robust to the nature of overyielding (if it is present), or the nature of the mean-variance scaling of species’ abundances. Consequently, it offers a promising framework for understanding the broad range of relationships between population and community variability observed in natural and experimental systems.

The fact that portfolio effects are commonly found even in the presence of increasing population variability suggests that synchrony tends to decrease with diversity in DSR studies, consistent with our conjecture that this pattern is likely to be common in nature. There have been only two empirical studies that explicitly quantify the diversity-dependence of synchrony, but both exhibit an asymptotic decline that is consistent with a small, positive mean correlation coefficient that remains approximately constant as diversity changes. Specifically, Roscher *et al*. (2011) found that synchrony decreased strongly with diversity, from a median of ~0.55 when *n* = 2 to ~0.1 when *n* = 60, which is similar to what would be expected for a community with 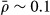 (cf. Fig. 2a, blue line). Similarly, Isbell *et al*. (2009) found that synchrony decreased from ~0.6 to ~0.3 as diversity increased from 2 to 8, consistent with 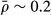. Of course, given that species are often added non-randomly along natural diversity gradients, and the idiosyncratic nature of species' responses to environmental fluctuations, there are likely to be exceptions to any general tendency for *ϕ* to decrease monotonically with diversity. An advantage of the framework in eqn 3 is that it makes no implicit simplifying assumptions about community structure that impose a particular functional form on this relationship.

The potential diversity-dependence of synchrony, along with eqn 3, offers an explanation for why studies of the DSR find a variety of different relationships between population and community variability. For instance, in a study of a natural diversity gradient among patches of boreal forest habitat, DeClerck *et al*. (2006) found that population variability increased with diversity, and between-species correlation coefficients were positive and large. The large correlation coefficients suggest that *ϕ* exhibited relatively little change with diversity in this system, because its value would have been dominated by the second term in eqn 8, which is independent of diversity. In this case, the DSR would be driven by how population variability changes with diversity, and, indeed, that appears to be precisely what happens: this study documented one of the very few empirical examples of an inverse portfolio effect in the literature. Conversely, in the experimental grassland communities of Roscher *et al*. (2011), synchrony was strongly diversity-dependent, so a portfolio effect could be apparent in spite of the fact that population variability increased with diversity.

Equation 7 extends the framework in eqn 3 to separate out the contribution of overyielding to population variability, and shows that overyielding and mean-variance scaling can have a broader range of effects than has been assumed in the literature (Figs and 4; Tilman *et al*. 2006; Isbell *et al*. 2009; Hector *et al*. 2010; Roscher *et al*. 2011), based on early analytical work suggesting that overyielding can induce a stabilising effect of diversity at the community level (Tilman 1999). Similarly, some empirical studies have reported *b* as a measure of the intensity of the DSR, with any value of *b* > 1 being taken as evidence that the portfolio effect is operating (e.g. Steiner *et al*. 2005; Polley *et al*. 2007; Isbell *et al*. 2009; Roscher *et al*. 2011), because simple analytical models have suggested that diversity should stabilise communities when the mean-variance scaling exponent *b* > 1, and destabilise them when *b* < 1 (Tilman *et al*. 1998; Tilman 1999; Loreau 2010). However, the derivation of these two conditions is sensitive to particular combinations of simplifying assumptions (see Appendix S5 in Supplementary Information). In fact, eqn 7 shows that, in general, the key determinants of the stabilising or destabilising effect of overyielding, at both population and community levels, are whether mean species abundance decreases (*x* > 0) or increases (*x* < 0) with diversity, and on whether species-level variances scale more (*b* > 2) or less (*b* < 2) than quadratically with the mean (Fig. 4). For instance, many empirical studies that have found both portfolio effects, and evidence of overyielding (i.e. *x* < 1), have invoked the latter as a key mechanism driving the former (Valone & Hoffman 2003; Tilman *et al*. 2006; Isbell *et al*. 2009; Hector *et al*. 2010; Roscher *et al*. 2011), based on early analytical work suggesting that overyielding can induce a stabilising effect of diversity at the community level (Tilman 1999). Similarly, some empirical studies have reported *b* as a measure of the intensity of the DSR, with any value of *b* > 1 being taken as evidence that the portfolio effect is operating (e.g. Steiner *et al*. 2005; Polley *et al*. 2007; Isbell *et al*. 2009; Roscher *et al*. 2011), because simple analytical models have suggested that diversity should stabilise communities when the mean-variance scaling exponent *b* > 1, and destabilise them when *b* < 1 (Tilman *et al*. 1998; Tilman 1999; Loreau 2010). However, the derivation of these two conditions is sensitive to particular combinations of simplifying assumptions (see Appendix S5 in Supplementary Information). In fact, eqn 7 shows that, in general, the key determinants of the stabilising or destabilising effect of overyielding, at both population and community levels, are whether mean species abundance decreases (*x* > 0) or increases (*x* < 0) with diversity, and on whether species-level variances scale more (*b* > 2) or less (*b* < 2) than quadratically with the mean (Fig. 3).

The strength of the portfolio effect is widely believed to be enhanced whenever evenness is greater, and this effect has been reproduced in the few theoretical studies that have relaxed the evenness assumption (Doak *et al*. 1998; Loreau 2010). This makes intuitive sense: if a community is dominated by one species, then adding rare species will produce only a small reduction in total community variance, which will be dominated by the abundant species' population variability. However, eqns 3 and 7 show how evenness may actually increase or decrease community stability, depending on its combined effects on synchrony and population variability. This context-dependence may help to explain why there is a lack of consistency in empirical relationships between stability, evenness and diversity (Steiner *et al*. 2005; Polley *et al*. 2007; van Ruijven & Berendse 2007; Isbell *et al*. 2009; Grman *et al*. 2010; Mikkelson *et al*. 2011). For instance, several empirical studies have found that, when averaged over all species in the community, *b* < 2 (i.e. larger populations are more stable: Bai *et al*. 2004; Leps 2004; Steiner *et al*. 2005; Polley *et al*. 2007; Roscher *et al*. 2011). This implies a stabilising effect of unevenness at the community level, although there is some evidence that species-specific deviations from the mean-variance scaling relationship may play an additional role (Grman *et al*. 2010: see Future Directions, below).

In decomposing population variability into a mean-abundance effect and a single-species variability effect, two assumptions were made that are more restrictive than those used in the derivation of our more general model unifying population and community variability: power-law scaling of the temporal mean and variance of abundance, and a monotonic change in mean abundance with diversity. As there is strong support for both such relationships in nature, and they are commonly examined in both theory and experiments of the DSR, these additional assumptions may seem, at first, to be relatively innocuous. However, their inclusion in eqn 7 implies the additional assumptions that the mean-variance scaling exponent and the overyielding coefficient are the same for all species in the community, and do not vary as functions of diversity. In nature, the extent to which these assumptions are violated varies between systems. For instance, Yang *et al*. (2011) found that a single mean-variance scaling exponent explained > 90% of the variation in temporal variances in alpine meadow communities (also see, e.g. Isbell *et al*. 2009), but van Ruijven & Berendse (2007), also studying herbaceous plants, found a nearly fourfold variation in the mean-variance scaling exponent among species. If mean-variance scaling exponents vary independently of species' relative abundances and responses to overyielding, then we would not expect the relationships shown in Fig. 3 to be qualitatively affected, and this is consistent with the results of preliminary simulations (not shown). However, covariation between mean-variance scaling parameters and species' relative abundances could change the way weighted average population variability changes with diversity. For instance, if disproportionately abundant species have lower than average *b*, then 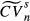 will tend to be smaller than predicted by eqn 7, because more stable species contribute disproportionately to the weighted average. There is empirical evidence for such relationships. For instance, Grman *et al*. (2010), examining residuals of an aggregate mean-variance scaling relationship, found evidence that disproportionately abundant species had smaller mean-variance scaling exponents than less abundant species.

Finally, although eqn 3 unifies population and community variability under a much broader range of conditions than previous models, it does retain one assumption of nearly all DSR theory that is likely to be violated to some degree, particularly in experimental manipulations of diversity gradients: that the community is fluctuating around a stochastic equilibrium (‘stationarity’). Few DSR studies explicitly address the stationarity assumption (see Tilman *et al*. 2006; Grman *et al*. 2010 for exceptions). Nevertheless, in most experimental diversity manipulations, stationarity is likely to be violated, at least to some degree. There are certainly some circumstances in which estimates of the portfolio effect could be biased by non-stationary dynamics. For example, if a competitively structured community begins with all species abundances well below, or well above, their equilibrium values, then most species will tend to increase or decrease, respectively, and exhibit much more synchronous dynamics, and higher species-level variances, than they would exhibit at equilibrium. Conversely, an assemblage that begins with some species well below, and others well above, their equilibrium values, dynamics may appear initially highly asynchronous relative to equilibrium, as over-abundant species persistently decline and under-abundant species increase towards their respective equilibria.

A comprehensive assessment of how these biases may influence empirical estimates of DSRs in nature is not possible, as few studies report evidence for or against underlying temporal trends in species abundances. However, two findings from recent meta-analyses suggest that experimental estimates of DSRs are unlikely to be consistently biased, relative to DSRs on natural diversity gradients. Firstly, one might expect shorter experiments to be more dominated by transient dynamics, but there does not appear to be any relationship between experiment duration and the effect of diversity on either population or community variability. Secondly, there are no significant differences in the mean effect sizes of DSR studies that involve direct diversity manipulations, indirect manipulations or that use natural diversity gradients (Campbell *et al*. 2011; also see Jiang & Pu 2009). However, studies of the DSR on natural diversity gradients do exhibit greater among-study variability than manipulative studies (Campbell *et al*. 2011). Thus, natural diversity gradients produce more instances in which portfolio effects do not occur (e.g. Rodriguez & Hawkins 2000; DeClerck *et al*. 2006), but they also produce instances of very strong portfolio effects (e.g. McNaughton 1985; Mouillot *et al*. 2005; Romanuk *et al*. 2009). This indicates that the non-random addition of species that occurs along natural diversity gradients adds more complexity to the community-level effects of diversity than may be apparent in experimental studies (Mittelbach *et al*. 2001; Huston & McBride 2002), and highlights the importance of having a framework for understanding DSRs that is robust to idiosyncratic changes in species’ mean abundances, variances and covariances with increasing diversity, such as eqn 3.

## Conclusions and Future Directions

The framework developed here makes explicit the relationships between several phenomena that previous theoretical and empirical studies have found to have important effects on the diversity–stability relationship, by relaxing several important simplifying assumptions that have been employed in various combinations in previous study. It makes explicit how the DSR depends on how two quantities change with diversity: the weighted average species-level variability (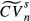), and community synchrony (*ϕ*). Moreover, it clarifies how the strength of overyielding (*x*), and the slope of the mean-variance scaling relationship (*b*), interact to influence population and community variability. Both species-level variability and synchrony depend on evenness, and, in most cases, are likely to vary as a function of diversity. This synthesis reveals important interactions between these different phenomena that influence the strength, and even the direction, of the DSR.

Our framework also suggests several particularly promising areas for further study. In particular, the synchrony index, *ϕ* is the key community property linking population and community variability. Only three empirical studies to date have explicitly estimated this quantity (Isbell *et al*. 2009; Roscher *et al*. 2011; Yang *et al*. 2011). However, virtually all empirical studies of the DSR collect the data necessary to estimate *ϕ*, meaning that a re-examination of existing data has the potential to rapidly flesh out our understanding of how *ϕ* changes with diversity in different types of assemblages. Similarly, population-level variability is universally understood to have a key influence on the DSR, but the way population variability is measured is inconsistent. Some studies quantify population variability separately by species; others compute (unweighted) averages across species at each diversity level; still others compute means and variances separately for all species at all diversity levels, and examine the aggregate relationship for systematic changes with diversity. Indeed, this inconsistency has been identified as a key barrier to our understanding of the relationship between population and community variability (Campbell *et al*. 2011). Equation 3 reveals that the critical measure of population variability, at least from the standpoint of the DSR, is a weighted average, 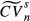. To date, no empirical studies of the DSR have measured population variability in this way (although most studies will have collected the data necessary to do so), suggesting that a re-examination of population variability and synchrony in empirical studies of the DSR may offer fresh insights into diversity–stability relationships in nature.

The development of portfolio effect theory by analysis of properties of the community covariance matrix (eqn 1b), or by analysis of community-dynamic models, have often been seen as mutually exclusive alternatives (Loreau 2010). However, statistical frameworks such as that proposed here can provide a common language for the interpretation and comparative analysis of studies of the DSR in both empirical and model communities. Community-dynamic models produce long-run means and variances of population and community abundance, and thus their outputs can be interpreted within the framework outlined here, just as empirical data can. Such approaches can reveal how particular assumptions about population dynamics and species interactions impose particular constraints on the diversity-dependence of community synchrony (Loreau 2010), and average population variability (Tilman 1999), as well as on the particular components of species-level variability, such as the nature of the mean-variance scaling relationship (e.g. Tilman 1999; Kilpatrick & Ives 2003).

## Supporting Information

As a service to our authors and readers, this journal provides supporting information supplied by the authors. Such materials are peer-reviewed and may be re-organised for online delivery, but are not copy-edited or typeset. Technical support issues arising from supporting information (other than missing files) should be addressed to the authors.
