# Supplementary material for: Understanding diversity–stability relationships: towards a unified model of portfolio effects
Source: Ecol Lett. 2012 Oct 24;16(2):140–50. doi: 10.1111/ele.12019 (PMC3588152; doi:10.1111/ele.12019)
Supplement: Supplementary file 1 [file ele0016-0140-sd1.pdf]

Appendix S1:

Effects of Unequal Variances on the Mean Correlation Coefficient and Synchrony Index

A major shortcoming of the mean correlation coefficient as a measure of community synchrony is that it is not robust to unequal variances. In contrast, the synchrony index ϕ implicitly accounts for the different effects of species with large versus small variances on total community variance. This can be illustrated with a simple toy example comparing two community covariance matrices, which differ only in the inequality of their variances.

Consider a community of *n* species, where the first *n*-1 species are perfectly correlated with each other ($\rho = 1$), and the nth species is perfectly negatively correlated with the other *n* species ($\rho = -1$). First, consider the case where all species have the same variance, *v*. Then, the community covariance matrix is:

$$\mathbf{V}_n = \begin{pmatrix} v & \dots & v & -v \\ \vdots & \ddots & \vdots & \vdots \\ v & \cdots & v & -v \\ -v & \cdots & -v & v \end{pmatrix} \tag{S1.1}$$

The synchrony index for this community (from eq. 2) is:

$$\phi = \frac{\sum_{ij} v_n^s(i,j)}{\left(\sum_i \sqrt{v_n^s(i,i)}\right)^2} = \frac{(n-1)^2 v + v - 2(n-1)v}{n^2 v} = \frac{(n-2)^2}{n^2} \tag{S1.2}$$

and the mean correlation coefficient is:

$$\bar{\rho} = \left(\frac{(n-1)n}{2}\right)^{-1} \left((1)\frac{(n-1)(n-2)}{2} + (-1)(n-1)\right) = \frac{n-4}{n} \tag{S1.3}$$

When there are only two species in the community (*n*=2), they are perfectly negatively correlated, and, consistent with this, $\phi = 0$ and $\bar{\rho} = -1$. As *n* increases, the negatively correlated species contributes a smaller and smaller portion of the total community variance, and the community variability is increasingly dominated by the perfectly synchronized species.

Accordingly, both the synchrony index and the mean correlation coefficient converge to 1 as $n \to \infty$.

Now consider a community with the same correlation coefficients, and where the first *n*-1 species still have variance *v*, but where the variance of the nth species is now $(n\text{-}1)^2 v$:

$$\mathbf{V}_n = \begin{pmatrix} v & \cdots & v & -(n-1)v \\ \vdots & \ddots & \vdots & \vdots \\ v & \cdots & v & -(n-1)v \\ -(n-1)v & \cdots & -(n-1)v & (n-1)^2 v \end{pmatrix} \qquad \text{(S1.4)}$$

Note that this community is perfectly asynchronous: the total community variance (sum of all of the covariance matrix elements) is zero (i.e., total community size remains constant over time), as long as there is more than one species in the community. This is reflected in the synchrony index, which is now zero for any $n>1$:

$$\phi = \frac{\sum_{ij} v_n^S(i,j)}{\left(\sum_i \sqrt{v_n^S(i,i)}\right)^2} = \frac{0}{4(n-1)^2 v} = 0 \qquad \text{(S1.5)}$$

In contrast, the mean correlation coefficient is the same as for the community with equal variances (eq. S1.3, above). In other words, even though this community is perfectly asynchronous (it remains constant over time), the mean correlation coefficient actually still converges to the opposite extreme (perfect synchrony, $\bar{\rho} = 1$) as species richness increases.

The problematic behavior of the mean correlation coefficient for the unequal community arises because the contribution of the negatively correlated species to the mean correlation is the same as the contribution of all the other species (and thus makes a proportionately smaller contribution at larger *n*), even though it contributes very disproportionately to the total variance of the community in the second example for all *n*.
