# Supplementary material for: Understanding diversity–stability relationships: towards a unified model of portfolio effects
Source: Ecol Lett. 2012 Oct 24;16(2):140–50. doi: 10.1111/ele.12019 (PMC3588152; doi:10.1111/ele.12019)
Supplement: Supplementary file 2 [file ele0016-0140-sd2.pdf]

Appendix S2: Derivation of Relationship between Population and Community Variability

To derive eq. (3), we re-arrange eq. (2) by solving for community variance:

$$v_n^c = \phi\left(\sum_i \sqrt{v_n^s(i)}\right)^2 \tag{S2.1}$$

We then divide both sides by temporal mean of total community abundance, m_n^c (i.e., the sum of mean abundances of all species in the community):

$$CV_n^c = \frac{\sqrt{v_n^c}}{m_n^c} = \frac{\sqrt{\phi\left(\sum_i \sqrt{v_n^s(i)}\right)^2}}{m_n^c} \tag{S2.2}$$

This allows us to express community variability as a dimensionless quantity: the coefficient of variation of total community abundance, CV_n^c. We then factor out the synchrony index:

$$CV_n^c = \frac{\sqrt{\phi\left(\sum_i \sqrt{v_n^s(i)}\right)^2}}{\sum_i m_n^s(i)} = \sqrt{\phi}\,\frac{\sum_i \sqrt{v_n^s(i)}}{\sum_i m_n^s(i)} \tag{S2.3}$$

Equation (3) says that community variability is simply the product of a synchrony effect, $\sqrt{\phi}$, and a population variability effect. One way to express the population variability is as the average standard deviation of species abundance, divided by the average mean species abundance. We can see this more clearly by dividing the right-hand numerator and denominator in (S2.3) by the number of species, *n*:

$$CV_n^c = \frac{\sqrt{\phi\left(\sum_i \sqrt{v_n^s(i)}\right)^2}}{\sum_i m_n^s(i)} = \sqrt{\phi}\,\frac{\frac{1}{n}\sum_i \sqrt{v_n^s(i)}}{\frac{1}{n}\sum_i m_n^s(i)} \tag{S2.4}$$

However, the link between population and community variability can be expressed more intuitively by recognizing that the ratio in eq. (S2.4) is equivalent to a weighted average of each species' coefficient of variation, where the weighting is by each species' relative mean abundance. We can do this by first pulling the sum in the numerator out in front of the fraction:

$$CV_n^c = \sqrt{\phi}\,\frac{\sum_i \sqrt{v_n^s(i)}}{\sum_i m_n^s(i)} = \sqrt{\phi}\sum_i \frac{\sqrt{v_n^s(i)}}{\sum_j m_n^s(j)}, \tag{S2.5}$$

and then multiplying through by ${m_n^s(i)}/{m_n^s(i)}$:

$$CV_n^c = \sqrt{\phi} \sum_i \frac{\sqrt{v_n^s(i)}}{\sum_j m_n^s(j)} = \sqrt{\phi} \sum_i \frac{m_n^s(i)}{\sum_j m_n^s(j)} \frac{\sqrt{v_n^s(i)}}{m_n^s(i)} = \sqrt{\phi}\, \widetilde{CV}_n^s \qquad \text{(S2.6)}$$

The term inside the sum in eq. (S2.6) is simply the relative mean abundance of species *i* (i.e., mean abundance relative to the mean total community abundance), times the coefficient of variation of abundance of species *i*. That is, the sum represents a weighted average species-level coefficient of variation, where the weighting is by species' relative abundance. Thus, for instance, in the special case where all species have the same mean abundance, $\widetilde{CV}_n^s$ collapses to a normal unweighted average CV.
