# Supplementary material for: Understanding diversity–stability relationships: towards a unified model of portfolio effects
Source: Ecol Lett. 2012 Oct 24;16(2):140–50. doi: 10.1111/ele.12019 (PMC3588152; doi:10.1111/ele.12019)
Supplement: Supplementary file 3 [file ele0016-0140-sd3.pdf]

Appendix S3: Derivation of Relationship between Mean-Abundance Effect and Population Variability

To derive eq (7) in the main text, we first rewrite eq. (4), using the mean-variance scaling relationship from eq. (5):

$$\widetilde{CV}_n^s = \frac{\sum_i \sqrt{v_n^s(i)}}{\sum_i m_n^s(i)} = \frac{\sum_i \sqrt{a\left(m_n^s(i)\right)^b}}{\sum_i m_n^s(i)} \qquad \text{(S3.1)}$$

Then, we substitute the right-hand side of eq. (6) for $m_n^s(i)$ in eq. (S3.1), and factor out the n^x terms in the resulting numerator and denominator:

$$\widetilde{CV}_n^s = \frac{\sum_i \sqrt{a\left(m_n^s(i)\right)^b}}{\sum_i m_n^s(i)} = \frac{\sum_i \sqrt{a\left(\frac{m_1(i)}{n^x}\right)^b}}{\sum_i \frac{m_1(i)}{n^x}} = \frac{\sqrt{\left(\frac{1}{n^x}\right)^b}}{\frac{1}{n^x}} \frac{\sum_i \sqrt{a\left(m_1(i)\right)^b}}{\sum_i m_1(i)}. \qquad \text{(S3.2)}$$

We then simplify the left-most fraction on the right-hand side of eq. (S3.2), to obtain:

$$\widetilde{CV}_n^s = \frac{n^{-\frac{xb}{2}}}{n^{-x}} \frac{\sum_i \sqrt{a\left(m_1(i)\right)^b}}{\sum_i m_1(i)} = \sqrt{n^{(2-b)x}} \frac{\sum_i \sqrt{a\left(m_1(i)\right)^b}}{\sum_i m_1(i)}. \qquad \text{(S3.3)}$$

Next, analogous to the derivation of $\widetilde{CV}_n^s$ (Appendix S2, eqs. S2.3-S2.5), we note that the fraction on the far right-hand side of eq. (S3.3) is equivalent to the weighted average of species' CV in monoculture:

$$\widetilde{CV}_n^s = \sqrt{n^{(2-b)x}} \frac{\sum_i \sqrt{a\left(m_1(i)\right)^b}}{\sum_i m_1(i)} = \sqrt{n^{(2-b)x}} \frac{\sum_i \sqrt{v_1(i)}}{\sum_i m_1(i)} = \sqrt{n^{(2-b)x}}\, \widetilde{CV}_1. \qquad \text{(S3.4)}$$

Substituting the right-hand side of the above equation into eq. (3) in the main text, we obtain eq. (7).
