# Supplementary material for: Understanding diversity–stability relationships: towards a unified model of portfolio effects
Source: Ecol Lett. 2012 Oct 24;16(2):140–50. doi: 10.1111/ele.12019 (PMC3588152; doi:10.1111/ele.12019)
Supplement: Supplementary file 4 [file ele0016-0140-sd4.pdf]

Appendix S4: Effect of Direct Interactions on Diversity-Dependence of Synchrony

To investigate the effect of incorporating direct interactions on the tendency for the synchrony index, ϕ, to decrease with diversity, we calculate synchrony from the analytical results of Ives *et al*. (1999) and Ives *et al.* (2003). Specifically, we consider the discrete-time Lotka-Volterra model of symmetric, diffuse competition:

$$N_i(t+1) = N_i(t) \cdot \exp\left[r\left(1 - \frac{N_i(t) + \alpha \sum_{j \neq i}^{n} N_j(t)}{K}\right)\right] \cdot \exp(\epsilon_i(t)) \tag{S4.1}$$

where $N_i(t)$ is population size of species i and time t, r =intrinsic growth rate, K =Carrying capacity, α = competition coefficient, and $\varepsilon_i(t)$ is a random perturbation to the growth rate due to environmental fluctuations (drawn for all n species in the community from a multivariate normal distribution with variances σ_e^2 and correlation coefficients ρ_e). Note that the ρ_e represent the correlations in species' responses to environmental fluctuations, which will, in general, be different from correlations in the overall fluctuations in abundance (ρ) used in the calculation of $\bar{\rho}$ and ϕ. Ives & Hughes (2002) derive a first-order Taylor series approximation of the model above:

$$n_i(t+1) = \left(1 - r\frac{N_i^*}{K}\right)n_i(t) - r\alpha\frac{N_i^*}{K}\sum_{j \neq i} n_j(t) + e_i(t) \tag{S4.2}$$

where $n_i(t)$ is abundance of species i, expressed as a perturbation from equilibrium at time t, N_i^* is the equilibrium population size of species i, and $e_i(t)$ is a re-scaled random perturbation to the growth rate ($e_i(t) = N_i^* \epsilon_i(t)$: Ives & Hughes 2002). Because this approximation is linear in N_i, the covariance matrix (eq. 1b) can be solved numerically (see eqs 15-17 in Ives *et al.* 2003).

From this, we can calculate the mean correlation coefficient, $\bar{\rho}$, and the synchrony index, as described in the main text.

Note that, when α =0, species are non-interacting, so $\bar{\rho}=\rho_e$ and ϕ exhibits the same pattern of diversity dependence as for the corresponding values of $\bar{\rho}$ in the main text (compare green lines in Fig. S4.1 with the orange, blue, and black lines in Fig. 2a). As α increases, the qualitative pattern of an asymptotic decline in ϕ is preserved (Fig. S4.1 compare different colored lines of the same line type). However, because competition makes species' dynamics less synchronous, ϕ decreases more rapidly, and to a lower value, compared to the equivalent non-interactive case, but the asymptotic shape of the decline is qualitatively unchanged (Fig. S4.1a).

LITERATURE CITED

Ives A.R., Dennis B., Cottingham K.L. & Carpenter S.R. (2003). Estimating Community Stability and Ecological Interactions From Time-Series Data. *Ecol. Monogr.*, 73, 301-330.

Ives A.R., Gross K., & Krug J.L. (1999). Stability and variability in competitive communities. *Science,* 286, 542-544.

Ives A.R. & Hughes J.B. 2002. General relationships between species diversity and stability in competitive systems. *Am. Nat.,* 159, 388-395.

Thibaut & Connolly Figure S4.1

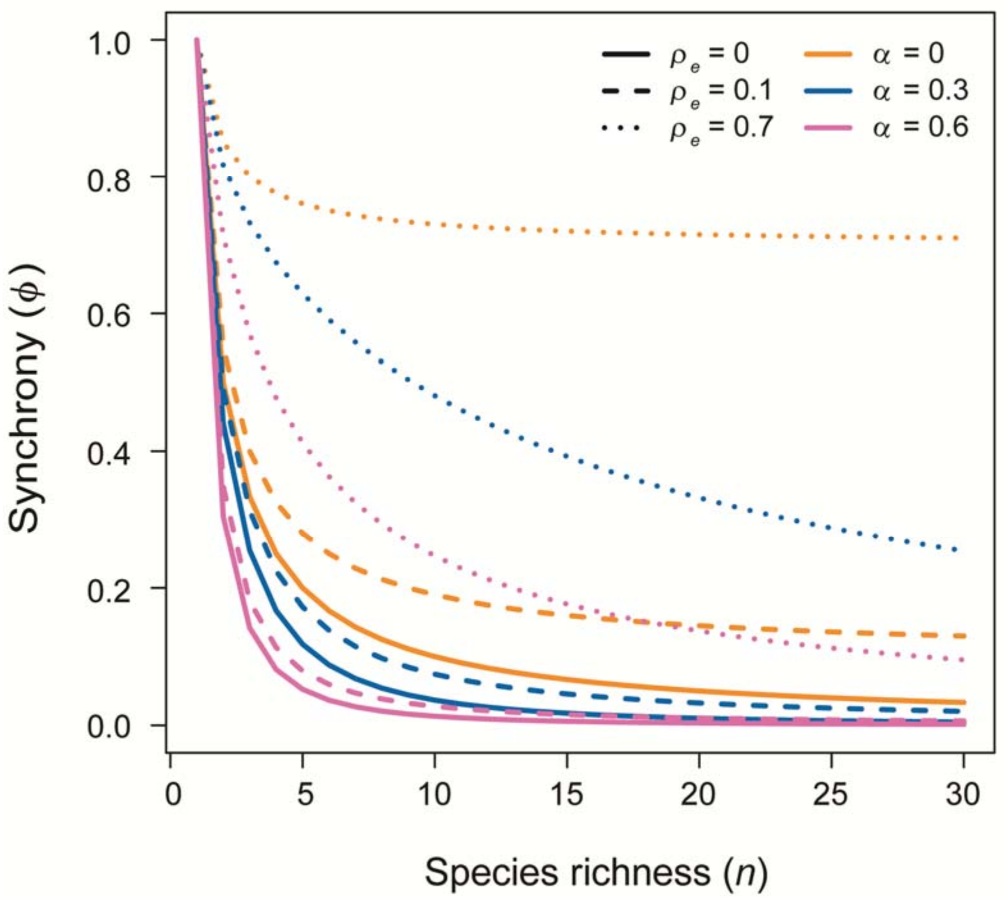

Figure S4.1. Diversity-dependence of the synchrony index under the discrete-time version of the Lotka-Volterra model, for the special case of symmetric, diffuse competition (eq. S4.1). For all

graphs, r=1, K=100, and α and ρ_e differ between lines as indicated on the figure panel. Note that, to better illustrate the asymptotic nature of the decline in synchrony, species richness is here plotted on an arithmetic scale (in contrast to the figures in the main text).
