# Supplementary material for: Understanding diversity–stability relationships: towards a unified model of portfolio effects
Source: Ecol Lett. 2012 Oct 24;16(2):140–50. doi: 10.1111/ele.12019 (PMC3588152; doi:10.1111/ele.12019)
Supplement: Supplementary file 5 [file ele0016-0140-sd5.pdf]

Appendix S5:

Overyielding, Porfolio Effects, and the "Threshold" of *b*=1

In Tilman's (1999) analytical model, $1 \leq b \leq 2$, $\rho=0$, and only values of $0 \leq x \leq 1$ are considered. For this special case, synchrony decreases inversely with diversity towards zero ($\phi=n^{-1}$ [eq. 8], black line in Fig. 2), and thus eq. (7) simplifies to:

$$CV_n^c = \widetilde{CV}_1 \sqrt{n^{(2-b)x}} \sqrt{n^{-1}}$$

Because $0<x<1$ and $1<b<2$, the mean-abundance effect increases with diversity, driving the increase in population variability with diversity (e.g., Fig. 3a, orange line). However, because it increases more slowly than synchrony decreases, there is still a portfolio effect (Fig. 4g). As overyielding increases (x decreases below 1), the increase in the mean-abundance effect with diversity becomes progressively weaker (Fig. 3a), and so the overall portfolio effect becomes stronger (Fig. 4g). Thus, overyielding promotes portfolio effects here by reducing the de-stabilization of species abundances, allowing the diversity-dependence of synchrony to dominate the community-level response.

These countervailing effects of overyielding and diversity-dependent synchrony are present in the experimental study of Roscher et al. (2011). They found $1<b<2$, and that community abundance increases, but species abundance decreases, with diversity (implying $0<x<1$). This should tend to cause population variability to increase with diversity. However, synchrony declines strongly with diversity towards a value close to zero (similar to what is implied by the $\rho=0$ assumption in Tilman's model). Consequently, at the community level, diversity-dependent synchrony outweighs the decreasing population stability, and a portfolio effect is observed.

Similarly, the threshold *b*=1 is derived from models sharing two key assumptions: constant community size, independent of diversity (i.e., *x*=1, no overyielding), and independence of species' fluctuations in abundance (i.e., all ρ=0). This corresponds to the special case above, with *x*=1:

$$CV_n^c = \widetilde{CV}_1 \sqrt{n^{(2-b)}} \sqrt{n^{-1}} = \widetilde{CV}_1 \sqrt{n^{(1-b)}}$$

Clearly, in this specific case, there is a portfolio effect when *b*>1, and an inverse portfolio effect when *b*<1. However, the result is highly sensitive to this particular combination of assumptions. Whenever either assumption is violated (as they will almost always be in nature), *b*=1 ceases to be a threshold defining portfolio effects.

LITERATURE CITED

Roscher C., Weigelt A., Proulx R., Marquard E., Schumacher J., Weisser W.W. et al. (2011). Identifying population- and community-level mechanisms of diversity–stability relationships in experimental grasslands. *J. Ecol.*, 99, 1460-1469

Tilman D. (1999). The ecological consequences of changes in biodiversity: a search for general principles. *Ecology*, 80, 1455-1474
